# Prognostic Factors and Survival Time in Patients with Small Bowel Tumors: A Retrospective Observational Study

**DOI:** 10.1155/2019/2912361

**Published:** 2019-05-02

**Authors:** Shokouh Taghipour Zahir, Zahra Heidarymeybodi, Sogol AleSaeidi

**Affiliations:** ^1^Shahid Sadoughi General Hospital, Shahid Sadoughi University of Medical Sciences, Yazd, Iran; ^2^Student Research Committee, Shahid Sadoughi General Hospital, Shahid Sadoughi University of Medical Sciences, Yazd, Iran

## Abstract

This study examines survival time in patients with small bowel tumors and determines its contributing factors. In this retrospective analytical study, the medical records of 106 patients with small bowel cancer (from 2006 to 2011) were investigated. The patients' data were extracted, including age, gender, clinical presentation, location of tumor, histological type, grade of tumor, site of metastasis, and type of treatment. The Kaplan-Meier test was used to estimate the overall survival time and the Log-rank test to compare the survival curves. The Cox regression was also used to evaluate the effect of the confounding variables on survival time. This study was conducted on 106 patients with a median age of 60 years (Min: 7, Max: 87). The tumor types included adenocarcinoma (n=78, 73.6%), MALToma (n=22, 20.8%), neuroendocrine tumors (n=4, 3.8%), and sarcoma (n=2. 1.8%). Grade 3 adenocarcinomas had a significantly lower survival time (HR: 1.48, 95% CI: 0.46-2.86; P=.001). Combined therapy (chemotherapy and surgery) vs. single-therapy (only surgery) had no significant effects on the survival of the patients with MALToma (5 vs. 3 months, 95% CI: 1.89-5.26; P=.06). There were no significant differences between the survival time in adenocarcinoma and MALToma (12 vs. 20 months, 95% CI: 6.24-24.76; P=.49). Tumor grade was the only independent prognostic factor that affected survival in adenocarcinoma. The patients diagnosed with MALToma in the study also had a poor prognosis, and the type of treatment had no significant effect on their survival.

## 1. Introduction

Primary Small Bowel Tumors (SBTs) are a rare type of malignancies that are known as an obscure gastrointestinal (GI) cancer. Although the small bowel is larger than the other parts of the GI tract (in terms of both length and surface) and is located between two common sites of the digestive neoplasm (the colon and the stomach), only about 3% to 6% of GI malignancies are SBTs [1–3]. SBTs are vague tumors with risk factors as well as prognostic factors that remain unidentified to date. SBTs are ambiguous for a number of reasons in addition to their scarcity. These reasons include limitations in diagnostic methods for reaching and detecting lesions in the small intestine and the fact that they have almost 40 subtypes. Adenocarcinoma, lymphoma, neuroendocrine tumors (NET), and sarcoma account for the majority SBTs [2, 4–6]. Considering the heterogeneity in their histological type, the treatments for SBTs vary, although surgery is the main treatment for resecting the tumor [7]. Although SBTs are very rare, a steady increase has been reported in their incidence over the past few decades for unknown reasons [8–11]. Improved diagnostic methods [12] and the consideration of SBTs in clinical approaches are two possible reasons for this slight increase in their incidence.

The factors attributed to the lower survival time in patients with SBTs are not well understood. The 5-year survival rate can vary from 83% in localized tumors to 43% in tumors with distant metastasis [13]. Some studies suggest different survival times for each type of SBT. These studies have shown that 5-year survival is highest in NET and lowest in sarcoma [11, 14]. To date, the patient's age and gender, clinical presentations, location of the tumor, histological type of the tumor, tumor grade, and stage and carcinoembryonic antigen (CEA) levels have been investigated as potential factors affecting the survival rate [14, 15].

The present study was conducted to estimate the overall survival time in SBTs and evaluate the effects of tumor type, type of treatment, histological grade, site of metastasis, and anatomical location on the survival of SBTs.

## 2. Materials and Methods

### 2.1. Study Population and Sample Collection

The present retrospective, observational, analytical study was conducted using the STROBE statement checklist. All the patients with small bowel malignancy referred to the pathology department of Shahid Sadoughi Hospital in Yazd, Iran, from March 2006 to March 2011, were examined in this study. The inclusion criterion for the study was primary (not metastatic) small bowel tumor. Of the 128 patients examined, 22 had metastatic tumors and were, therefore, excluded, leaving 106 patients for the research. All the patients were followed up from their date of referral to the end of 2016.

### 2.2. Ethics Approval

All procedures performed in studies involving human participants were in accordance with the ethical standards of Shahid Sadoughi General Hospital research committee and with the 1964 Helsinki Declaration and its later amendments or comparable ethical standards.

### 2.3. Data Collection and Reporting

The biopsy slides were extracted from the patients' records and were reviewed by experienced pathologists. The patients' demographic and clinical characteristics were also retrieved from the hospital records by data collection forms that inquired about the patient's age, gender, being outpatient/inpatient, disease symptoms, anatomical location of the tumor, region of metastasis, and type of treatment. The researchers followed up the patients to identify their survival status and time and cause of death (if applicable) by referring to their hospital records. If their survival status was not detectible by the researchers, the patients were considered missing subjects. The tumors diagnosed as SBTs are classified into four subtypes, including adenocarcinoma, lymphoma (MALT), NET, and sarcoma (gastrointestinal stromal tumors (GIST) and leiomyosarcoma). Histological grade is reported as grade 1 (well differentiated), grade 2 (moderately differentiated), and grade 3 (poorly differentiated). The tumor subtype and histological grade were categorized based on the WHO classification.

### 2.4. Statistical Analysis

The median and range were reported for the continuous variables due to their skewness. The Kaplan-Meier test was used to estimate the overall survival time and the Log-rank test to compare the survival curves. Survival time was taken as the number of months the patient has lived after the treatment until the end of the follow-up. The Cox proportional hazard regression was used to analyze the prognostic factors, and the results of the analysis were reported with the hazard ratio (HR) and a 95% confidence interval (CI). The multivariate Cox regression was used for the covariates that had been proven statistically significant in the univariate model and the independent risk factors affecting survival were thus identified. For the Cox regression, age was divided into three categories (<50 years, 50-75, and 75>) in order to be fitted in the model. The patients with a missing survival status were excluded from the survival analysis. All the* P* values less than 0.05 were considered statistically significant. The analyses were carried out in IBM SPSS-22 for Windows (IBM Corp., Armonk, NY).

## 3. Results

### 3.1. Demographics Features and General Characteristics

The study was conducted on 106 patients (90 inpatients and 16 outpatients) with SBTs. Among them, 56.6% (n=60) were male and 43.4% (n=46) were female. The median age at the time of diagnosis was 60 and the age range was 7-87 years. The median follow-up duration was seven years (range: 5-10 years). A total of 96% of the patients presented with a loss of appetite, weight loss, fever, anemia, and growth retardation. More than half of the patients (64%) suffered from stomach ache, burning ache in the upper stomach, and a feeling of fullness. Constipation, diarrhea, vomiting, hematemesis, bloody stool, and dark urine were less common in the patients. A total of 78 (73.6%) of the tumors were adenocarcinoma, 22 (20.8%) were lymphoma, four (3.8%) were NET, and two (1.8%) were sarcoma, including one GIST and one leiomyosarcoma. Of the four NETs, two were in the ileum, one in the duodenum, and one in the jejunum. None of the NETs had metastasized and none were grade 3 (three NETs were in grade 2 and one was in grade 1). Both sarcoma cases were diagnosed in grade 3 and were found in the jejunum. GIST had a peritoneal metastasis and leiomyosarcoma had no metastasis.

### 3.2. Adenocarcinoma

Table 1 presents a summary of the clinical-pathological characteristics of the adenocarcinoma cases. None of the patients diagnosed with adenocarcinoma had any known underlying diseases such as familial polyposis or Crohn's disease. Of the 78 patients diagnosed with adenocarcinoma, 37 died due to this tumor, four died for nonrelated reasons, nine remained alive, and the survival status of 28 patients was missing. The effect of the patients' age, gender and tumor grade, location, type of treatment, and metastasis on survival was also evaluated. In the multivariate Cox regression, grade 3 (HR: 1.48, 95%CI: 0.46-2.86; P=.001) was the only factor independently affecting survival, as shown in Table 2.

### 3.3. Lymphoma

**Figure 1:**
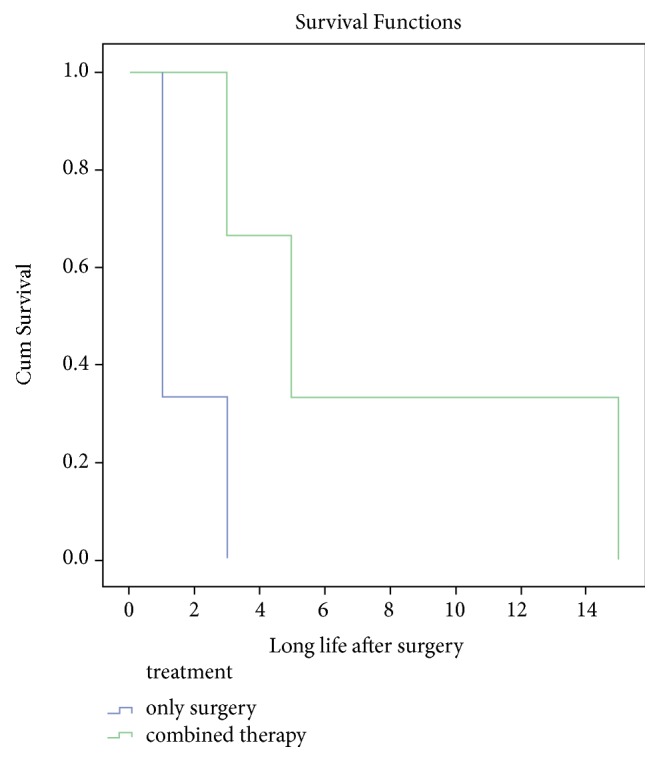
Kaplan-Meier curves illustrating overall survival of patients with MALToma who received chemotherapy after surgery (n =7) compared with patients who just had surgery (n =5).

**Figure 2:**
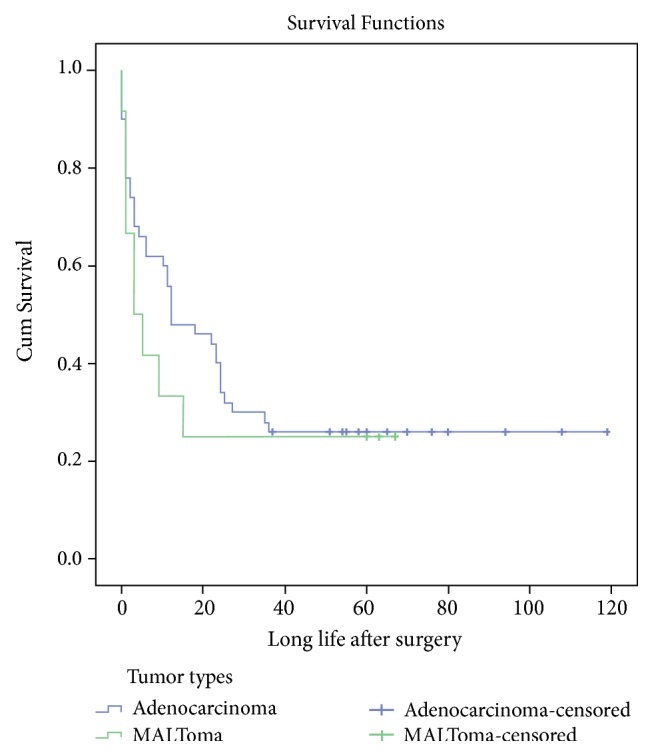
Kaplan-Meier curve illustrating overall survival of adenocarcinoma (n=78) compared with other types of tumors (n=22).

Of the 22 lymphomas included in this study, all were MALT lymphoma (MALToma). A past history of celiac disease was not reported in any of the patients. Nine of the MALTomas were found in the duodenum, eight in the ileum, and four in the jejunum. Omentum was the location of only one MALToma. Five patients had undergone only surgery and three only had chemotherapy. Combined therapy (chemotherapy plus surgery) was used in seven patients, while the others had not received any treatment at all. Death due to MALToma occurred in nine patients. Three patients remained alive until the end of the follow-up, and the survival status of the others was missing. The type of treatment (combined therapy vs. only surgery) had no effect on survival time in the patients diagnosed with MALToma (Figure 1 and 5 vs. 3 months, 95% CI: 1.89-5.26; P=.06). There were no significant differences between the median survival time in the adenocarcinoma cases compared to the MALToma cases (Figure 2 and 12 vs. 20 months, 95% CI: 6.24-24.76; P=.49).

## 4. Discussion

SBTs are a rare incidence and their risk factors as well as prognostic factors remain poorly understood. The review of the literature showed few studies on the risk factors affecting survival in SBTs. The present retrospective study was carried out to find the factors affecting the prognosis of SBTs and their patients' survival time. The results showed that grade 3 tumors independently affect survival in patients with adenocarcinoma and there is no significant difference between the survival of patients with adenocarcinoma and that of patients with MALToma.

Several hypotheses explain the reasons for the scarcity of small intestine malignancies, including the higher motility rate (peristaltic contraction) of the small intestine, which decreases the time of carcinogenic exposure, the lower generation of reactive oxidative species (ROS) that protect against lymphoma by increasing the level of Immunoglobulin A, and the lower bacterial population that reduces bile acid breakdown and thereby the potential carcinogen concentration as well [7]. Due to the correlation between colon cancer and small intestine cancer, it seems reasonable to consider the same risk factors for SBT as colon cancer. Risk factors can be divided into three categories, including background diseases and medical conditions (e.g., inflammatory bowel disease, celiac disease, and small intestine adenomas or familial adenomatous polyposis), other cancers (hereditary nonpolyposis colorectal cancer, sporadic colorectal cancer), and behavioral and environmental risk factors (alcohol consumption and cigarette smoking, a diet rich in red meat, obesity, and high BMI)[7, 16].

Since the clinical presentations of SBTs are often nonspecific, various diseases should be considered in their differential diagnosis. Diseases that are considered in their differential diagnosis are divided into metastatic and nonneoplastic diseases. Nonneoplastic diseases include the Peutz-Jeghers syndrome and Brunner's gland adenoma. Metastatic diseases can further be subclassified as intraperitoneal metastasis (such as mucinous tumor in the ovary and colon), hematogenous metastasis (such as in renal cell carcinoma, breast carcinoma, and malignant melanoma), and direct extension metastasis (such as biliary and colon cancer)[17]. The whole small intestine can be accessed by capsule endoscopy and double balloon endoscopy, and the chances of detecting SBTs at their early stages increase. GI bleeding without a known etiology often raises suspicion for SBTs (especially in younger patients). Almost 9% of the patients who had undergone capsule endoscopy for gastrointestinal bleeding were diagnosed with SBTs [4, 12, 18, 19]. In this study, most of the patients had nonspecific symptoms such as the loss of appetite and weight loss, and gastrointestinal bleeding (such as blood in vomit and stool) was observed in a limited number of the patients.

Adenocarcinoma is the most common subtype of SBTs. Adenocarcinoma cannot be detected at early stages of the disease and its late diagnosis makes for a very poor prognosis [20]. In line with previous studies, the prognosis of adenocarcinoma was also poor in the present study and the median survival time was only one year. Cardoso et al. [21] reported that adenocarcinoma has the lowest survival time among all the subtypes, but according to the present study, adenocarcinoma does not have a significantly lower survival time than lymphoma. This variation may be explained by the different sample size of the two studies. The present study compared 78 adenocarcinomas with 22 MALTomas; meanwhile, Cardoso et al. [21] examined only 11 adenocarcinomas and compared them with other small bowel tumors (n=18). In a study conducted by Chaiyasate et al. [22], as in line with the present findings, histological grade was found to be a predictive factor for survival. These researchers also found that tumor stage at the time of diagnosis, lymph node metastasis, and resection margins could also affect survival time in patients with small bowel adenocarcinoma. In disagreement with the present findings, Telmonti et al. [14] found that histological grade has no effect on the survival of patients with SBTs. They actually found that the stage and complete resection of the tumor are correlated with survival rather than grade, anatomical location, and age. In this study, age and anatomical location had no significant relationships with the patients' survival. The hypothesis is that since the present study did not evaluate the effect of tumor stage on survival, the prognostic effect of tumor grade becomes more prominent. The aforementioned study [14] showed that total tumor resection improved the patients' survival; however, there was no proof for the benefits of chemotherapy after surgery. In line with this report, the present findings showed that there was no significant difference in survival between the patients that undergone chemotherapy after surgery and the patients who only had surgery. The present study examined the effect of all these factors on adenocarcinoma, and 73% of the tumors in the study were adenocarcinoma.

Intestinal lymphoma accounts for 15-20% of all small bowel neoplasms. Small intestine lymphoma is widely heterogeneous, which means that lymphoma has subtypes such as MALT, DLBCL, EATL, and MCL. Poorer treatment outcomes have been reported for intestinal lymphoma compared to gastric lymphoma [23]. In a study comparing the subtypes of intestinal lymphoma, a better prognosis was reported for MALToma [24]. All the lymphomas included in the present study were MALTomas; therefore, comparing the results with findings on the other subtypes of lymphoma was not possible. The rate of survival was poor in the patients with MALToma, unlike another study conducted in Japan [24]; this disparity may be due to the lower socioeconomic status of the patients in the present study or the lower budget of the Iranian health care system. Treatment for intestinal lymphoma varies depending on histological type, age of the patient, and disease burden and may consist of surgery or chemotherapy or a combination of both. Some studies have reported benefits from radiotherapy for localized lymphoma [23]. In the study by Hong et al. [25], combined therapy (surgery and chemotherapy) was found to improve the survival of patients with lymphoma compared to chemotherapy alone or surgery alone. Conversely, in the present study, no improvements were observed in the survival of patients with MALToma between those who received combined therapy and those who received single therapy (surgery alone). One reason for this disparity of findings may be that the survival curves were evaluated in a limited number of patients with MALToma in this study.

Several studies have discussed the correlation between anatomical location and histological type of tumor. Adenocarcinoma has mostly been observed in the duodenum, lymphoma in the ileum, and GIST in the jejunum [16, 20, 23]. In the present study, the most common site for adenocarcinoma was the duodenum, and MALToma was also most commonly found in the duodenum, followed by the ileum. Only one jejunal GIST was observed in the present study.

The limitations of this study included the retrospective design and also the single-center data, which reduces the external validity of the findings. The sample size for survival analysis was also relatively small and this limitation could affect the outcome of the analysis. Also, since the pathology department had not received adequate data, assessing the tumor stage was impossible. Lastly, the researcher did not know which chemotherapy regimens were used for adenocarcinoma and lymphoma in the patients.

Since the small intestine adenocarcinoma is rare cancer, the question is does postsurgical chemotherapy change the prognosis of patients in stage I-III or not? And also does the use of fluoropyrimidine-based adjuvant therapy (FP), with or without oxaliplatin (Ox), can be effective? For these questions, the international phase III clinical trial (GLOBAL BALLAD) is underway [26].

In conclusion, adenocarcinoma was the most common SBT diagnosed in the single center that was examined in this study. The outcome of the patients with adenocarcinoma was not significantly worse compared to the patients with MALToma. Grade 3 adenocarcinomas have a worse prognosis and death due to cancer occurring earlier with this grade. The type of treatment had no significant effects on the survival of the patients with adenocarcinoma or MALToma. Although these tumors were found in older ages, they were also reported in children. The clinical features of all the tumor types were nonspecific, which demonstrates the need for considering SBTs in clinical approaches so as to avoid misdiagnosis.

## Figures and Tables

**Table 1:** Clinical-pathological characteristics of adenocarcinoma.

Factors	Subgroup	Number (%)

Sex	Male	44(56.4%)
	Female	34(43.6%)

Age (range: 24-87)	<50	12(15.4%)
	50-77	20(25.6%)
	>75	46(59.0%)

Anatomical location	Duodenum	35(44.9%)
	Jejunum	16(20.5%)
	Ileum	25(32.1%)
	Omentum	2(2.6%)

Metastasis	None	56(71.8%)
	Peritoneal	13(16.7%)
	Liver	5(6.4%)
	Lymphatic	4(5.1%)

Histological grade	Grade1	13(16.7%)
	Geade2	45(57.7%)
	Grade3	20(25.6%)

Type of treatment	None	24(22.6%)
	Chemotherapy	2(2.8%)
	Surgery	55(51.9%)
	Chemotherapy and surgery	24(22.6%)

**Table 2:** Univariate and multivariate Cox regression model.

	Univariate analysis			Multivariate analysis		
Factors	HR^1^	95%CI^2^	*P* value	HR	95%CI	*P* value

*Sex*						
Female	Reference					
Male	1.55	0.669-3.629				
*Age*						
<50	Reference					
50-75	0.247	0.058-1.048	0.058			
>75	0.793	0.310-2.032	0.629			
*Location*						
Duodenum	Reference					
Jejunum	0.081	0.014-0.0462	0.003	1.125	0.23-2.146	0.948
Ileum	0.098	0.016-0.584	0.038	0.226	0.36-1.432	0.114
*Metastasis*						
No	Reference					
Yes	0.940	0.391-2.259	0.889			
*Grade*						
1	Reference					
2	0.266	0.111-0.634	0.003	0.123	0.049-1.310	0.078
3	0.128	0.040-0.407	0.001	1.148	0.460-2.863	0.001
*Treatment*						
None	Reference					
Surgery	1.203	0.123-4.117	0.874			
Chemotherapy and surgery	0.658	0.051-6.419	0.738			

## Data Availability

The data used to support the findings of this study are available from the corresponding author upon request.
